# 2-(6-Chloro-2,3,4,9-tetra­hydro-1*H*-carbazol-1-yl­idene)propane­dinitrile

**DOI:** 10.1107/S1600536811046575

**Published:** 2011-11-12

**Authors:** M. Sekar, R. Velmurugan, A. V. Vijayasankar, P. Ramesh, M. N. Ponnuswamy

**Affiliations:** a Post Graduate and Research Department of Chemistry, Sri Ramakrishna Mission Vidyalaya College of Arts and Science, Coimbatore 641 020, India; b Department of Engineering Chemistry, Christ University, Bangalore 560 029, Karnataka, India; c Centre of Advanced Study in Crystallography and Biophysics, University of Madras, Guindy Campus, Chennai 600 025, India

## Abstract

The mol­ecular conformation of the title compound, C_15_H_10_ClN_3_, is stabilized by an intra­molecular N—H⋯N hydrogen bond with an *S*(7) ring motif. The crystal packing is controlled by N—H⋯N and C—H⋯N inter­molecular inter­actions. One of the methyl­ene groups of the cyclo­hexene ring is disordered over two positions with refined occupancies of 0.457 (12) and 0.543 (12).

## Related literature

For the biological activity of carbazole derivatives, see: Shufen *et al.* (1995 ▶); Magnus *et al.* (1992 ▶); Abraham (1975 ▶); Saxton (1983 ▶); Phillipson & Zenk (1980 ▶); Kirtikar & Basu (1933 ▶). For hydrogen-bond motifs, see: Bernstein *et al.* (1995 ▶).
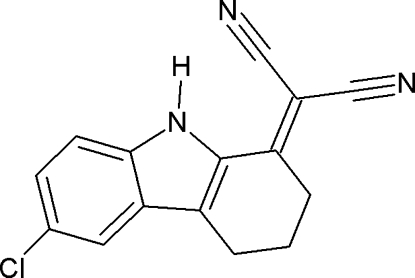


## Experimental

### 

#### Crystal data

C_15_H_10_ClN_3_
*M* *_r_* = 267.71
Monoclinic, 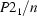

*a* = 7.5731 (3) Å
*b* = 7.6865 (3) Å
*c* = 22.2867 (8) Å
β = 97.437 (2)°
*V* = 1286.41 (9) Å^3^
*Z* = 4
Mo *K*α radiation
μ = 0.29 mm^−1^
*T* = 293 K
0.20 × 0.19 × 0.17 mm

#### Data collection

Bruker SMART APEX CCD detector diffractometer
Absorption correction: multi-scan (*SADABS*; Bruker, 1998 ▶) *T*_min_ = 0.945, *T*_max_ = 0.953
24123 measured reflections
3828 independent reflections
2569 reflections with *I* > 2σ(*I*)
*R*_int_ = 0.031

#### Refinement

*R*[*F* ^2^ > 2σ(*F* ^2^)] = 0.053
*wR*(*F* ^2^) = 0.164
*S* = 1.01
3828 reflections
186 parameters
1 restraint
H atoms treated by a mixture of independent and constrained refinement
Δρ_max_ = 0.42 e Å^−3^
Δρ_min_ = −0.30 e Å^−3^

### 

Data collection: *SMART* (Bruker, 1998 ▶); cell refinement: *SAINT-Plus* (Bruker, 1998 ▶); data reduction: *SAINT-Plus*; program(s) used to solve structure: *SHELXS97* (Sheldrick, 2008 ▶); program(s) used to refine structure: *SHELXL97* (Sheldrick, 2008 ▶); molecular graphics: *ORTEP-3* (Farrugia, 1997 ▶); software used to prepare material for publication: *SHELXL97* and *PLATON* (Spek, 2009 ▶).

## Supplementary Material

Crystal structure: contains datablock(s) global, I. DOI: 10.1107/S1600536811046575/bt5656sup1.cif

Structure factors: contains datablock(s) I. DOI: 10.1107/S1600536811046575/bt5656Isup2.hkl

Supplementary material file. DOI: 10.1107/S1600536811046575/bt5656Isup3.cml

Additional supplementary materials:  crystallographic information; 3D view; checkCIF report

## Figures and Tables

**Table 1:** Hydrogen-bond geometry (Å, °)

*D*—H⋯*A*	*D*—H	H⋯*A*	*D*⋯*A*	*D*—H⋯*A*
N1—H1⋯N16	0.94 (3)	2.60 (3)	3.373 (2)	139.5 (19)
N1—H1⋯N16^i^	0.94 (3)	2.27 (3)	3.099 (3)	147 (2)
C11—H11⋯N18^ii^	0.93	2.48	3.352 (3)	156

Symmetry codes: (i) 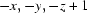; (ii) 
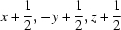
.

**Fig. 1.**
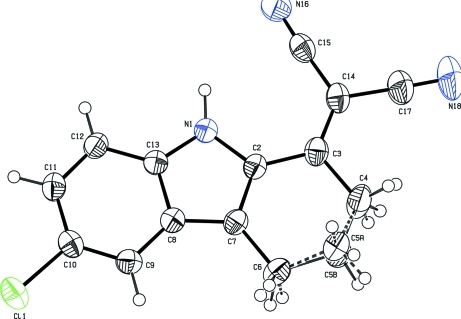
The molecular structure of the title compound, showing the atomic numbering and displacement ellipsoids drawn at the 50% probability level.

**Fig. 2.**
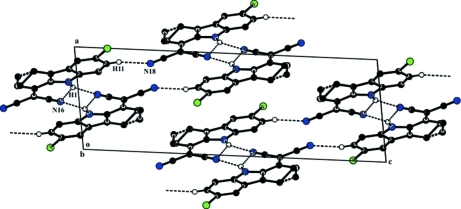
The crystal packing of the title compound. H atoms not involved in hydrogen bonding (dashed lines) have been omitted for clarity.

## supplementary crystallographic information

### Comment

Carbazole alkaloids obtained from natural sources have been the subject of extensive research, mainly because of their widespread applications in traditional medicine (Kirtikar & Basu, 1933). Aminocarbazoles are widely used as intermediates for the preparation of carbazole-based synthetic dyes, agrochemicals, pharmaceuticals, light-sensitive materials (Shufen *et al.*, 1995). Tetrahydrocarbazole systems are present in the framework of a number of indole-type alkaloids of biological interest (Magnus *et al.*, 1992; Abraham, 1975; Saxton, 1983; Phillipson *et al.*, 1980). Against this background and to ascertain the molecular structure and conformation, the crystal structure determination of the title compound has been carried out.

The *ORTEP* plot of the molecule is shown in Fig. 1. One of the C atoms of the cyclohexene ring is disordered with refined occupancies of 0.457 (12) and 0.543 (12). The sum of the bond angles around N1 [359.8°] is in accordance with *sp^2^* hybridization. The bond lengths (C15—N16) 1.145 (3)Å & (C17—N18) 1.144 (3)Å and the bond angles, (C14—C15—N16) 176.9 (2)° & (C14—C17—N18) 178.6 (3)° show linear character of the cyano group, a feature observed in carbonitrile compounds.

The crystal packing reveals that symmetry-related molecules are linked through a network by C—H···N, N—H···N and π···π types of intra and intermolecular interactions. The intramolecular N1—H1···N16 hydrogen bond generates a S(7) ring motif. The molecules at (*x*, *y*, *z*) and (-*x* - 1, -*y* - 1, -*z*) are linked by N1—H1···N16 hydrogen bonds into cyclic centrosymmetric *R*_2_^2^(14) dimer. The dimers are linked *via* inter molecular C11—H11···N18 hydrogen bond, which forms a one dimensional chain running along diagonally in *ac*-disection.

### Experimental

A mixture of 6-Chloro-1-oxo-1,2,3,4-tetrahydrocarbazole (7.5 mmol), and melanonitrile (7.5 mmol), ammonium acetate (0.57 g, 8.125 mmol) and acetic acid (1.5 ml, 24.75 mmol) in 12.5 ml of toluene was stirred at 105°C for 5 h. On cooling the precipitate that formed was filtered off, washed with hexane (20 ml) and dried at 100°C to give a crude product of 6-chloro-2-(1,2,3,4- tetrahydro-9*H*-carbazol-1-ylidene)propanedinitrile.The crystals of the title compound suitable for single XRD analysis were obtained by the slow evaporation method by using dichloroethane as solvent at room temperature.

### Refinement

The N-bound H atom was located in a difference Fourier map and refined isotropically. C-bound H atoms were positioned geometrically (C–H = 0.93–0.97 Å) and allowed to ride on their parent atoms, with *U*_iso_(H) = 1.2*U*_eq_(C) for all H atoms. One of the methylene groups of the cyclohexene ring is disordered over two positions with refined occupancies of 0.457 (12) and 0.543 (12).

### Crystal data

**Table:**

C_15_H_10_ClN_3_	*F*(000) = 552
*M**_r_* = 267.71	*D*_x_ = 1.382 Mg m^−^^3^
Monoclinic, *P*2_1_/*n*	Mo *K*α radiation, λ = 0.71073 Å
Hall symbol: -P 2yn	Cell parameters from 4023 reflections
*a* = 7.5731 (3) Å	θ = 2.8–30.5°
*b* = 7.6865 (3) Å	µ = 0.29 mm^−^^1^
*c* = 22.2867 (8) Å	*T* = 293 K
β = 97.437 (2)°	Block, brown
*V* = 1286.41 (9) Å^3^	0.20 × 0.19 × 0.17 mm
*Z* = 4	

### Data collection

**Table:**

Bruker SMART APEX CCD detector diffractometer	3828 independent reflections
Radiation source: fine-focus sealed tube	2569 reflections with *I* > 2σ(*I*)
graphite	*R*_int_ = 0.031
ω scans	θ_max_ = 30.5°, θ_min_ = 2.8°
Absorption correction: multi-scan (*SADABS*; Bruker, 1998)	*h* = −10→10
*T*_min_ = 0.945, *T*_max_ = 0.953	*k* = −10→10
24123 measured reflections	*l* = −30→30

### Refinement

**Table:**

Refinement on *F*^2^	Primary atom site location: structure-invariant direct methods
Least-squares matrix: full	Secondary atom site location: difference Fourier map
*R*[*F*^2^ > 2σ(*F*^2^)] = 0.053	Hydrogen site location: inferred from neighbouring sites
*wR*(*F*^2^) = 0.164	H atoms treated by a mixture of independent and constrained refinement
*S* = 1.01	*w* = 1/[σ^2^(*F*_o_^2^) + (0.0867*P*)^2^ + 0.2888*P*] where *P* = (*F*_o_^2^ + 2*F*_c_^2^)/3
3828 reflections	(Δ/σ)_max_ < 0.001
186 parameters	Δρ_max_ = 0.42 e Å^−^^3^
1 restraint	Δρ_min_ = −0.30 e Å^−^^3^

### Special details

**Table:**

Geometry. All e.s.d.'s (except the e.s.d. in the dihedral angle between two l.s. planes) are estimated using the full covariance matrix. The cell e.s.d.'s are taken into account individually in the estimation of e.s.d.'s in distances, angles and torsion angles; correlations between e.s.d.'s in cell parameters are only used when they are defined by crystal symmetry. An approximate (isotropic) treatment of cell e.s.d.'s is used for estimating e.s.d.'s involving l.s. planes.
Refinement. Refinement of *F*^2^ against ALL reflections. The weighted *R*-factor *wR* and goodness of fit *S* are based on *F*^2^, conventional *R*-factors *R* are based on *F*, with *F* set to zero for negative *F*^2^. The threshold expression of *F*^2^ > σ(*F*^2^) is used only for calculating *R*-factors(gt) *etc*. and is not relevant to the choice of reflections for refinement. *R*-factors based on *F*^2^ are statistically about twice as large as those based on *F*, and *R*- factors based on ALL data will be even larger.

### Fractional atomic coordinates and isotropic or equivalent isotropic displacement parameters (Å^2^)

**Table:**

	*x*	*y*	*z*	*U*_iso_*/*U*_eq_	Occ. (<1)
Cl1	0.49620 (8)	0.90952 (7)	0.60857 (3)	0.0642 (2)	
N1	0.15258 (19)	0.3281 (2)	0.46981 (6)	0.0405 (3)	
H1	0.102 (3)	0.225 (3)	0.4828 (11)	0.067 (7)*	
C2	0.1477 (2)	0.3928 (2)	0.41129 (7)	0.0390 (4)	
C3	0.0745 (2)	0.3114 (2)	0.35585 (8)	0.0439 (4)	
C4	0.0935 (4)	0.4150 (3)	0.29963 (9)	0.0653 (6)	
H4A	−0.0054	0.3869	0.2690	0.078*	0.457 (12)
H4B	0.2021	0.3792	0.2843	0.078*	0.457 (12)
H4C	0.1690	0.3487	0.2760	0.078*	0.543 (12)
H4D	−0.0232	0.4214	0.2760	0.078*	0.543 (12)
C6	0.2437 (3)	0.6641 (3)	0.35882 (8)	0.0513 (5)	
H6A	0.3595	0.6447	0.3458	0.062*	0.457 (12)
H6B	0.2335	0.7866	0.3682	0.062*	0.457 (12)
H6C	0.3688	0.6823	0.3555	0.062*	0.543 (12)
H6D	0.1891	0.7769	0.3628	0.062*	0.543 (12)
C7	0.2261 (2)	0.5572 (2)	0.41368 (8)	0.0404 (4)	
C8	0.2803 (2)	0.5957 (2)	0.47570 (8)	0.0391 (4)	
C9	0.3633 (2)	0.7404 (2)	0.50607 (8)	0.0443 (4)	
H9	0.3960	0.8370	0.4849	0.053*	
C10	0.3943 (2)	0.7335 (2)	0.56802 (8)	0.0449 (4)	
C11	0.3465 (2)	0.5898 (2)	0.60126 (8)	0.0454 (4)	
H11	0.3699	0.5911	0.6433	0.054*	
C12	0.2654 (2)	0.4470 (2)	0.57250 (8)	0.0437 (4)	
H12	0.2338	0.3514	0.5943	0.052*	
C13	0.2319 (2)	0.4504 (2)	0.50921 (8)	0.0381 (4)	
C14	−0.0043 (3)	0.1511 (3)	0.35074 (8)	0.0476 (4)	
C15	−0.0238 (3)	0.0361 (3)	0.40008 (9)	0.0498 (5)	
N16	−0.0422 (3)	−0.0613 (2)	0.43766 (9)	0.0659 (5)	
C17	−0.0748 (4)	0.0825 (3)	0.29288 (11)	0.0693 (6)	
N18	−0.1317 (4)	0.0246 (4)	0.24718 (11)	0.1157 (10)	
C5A	0.0989 (7)	0.6133 (7)	0.3089 (3)	0.0420 (16)	0.457 (12)
H5A	0.1198	0.6697	0.2716	0.050*	0.457 (12)
H5B	−0.0153	0.6527	0.3190	0.050*	0.457 (12)
C5B	0.159 (2)	0.5788 (15)	0.3049 (4)	0.223 (8)	0.543 (12)
H5C	0.0610	0.6539	0.2894	0.268*	0.543 (12)
H5D	0.2447	0.5860	0.2761	0.268*	0.543 (12)

### Atomic displacement parameters (Å^2^)

**Table:**

	*U*^11^	*U*^22^	*U*^33^	*U*^12^	*U*^13^	*U*^23^
Cl1	0.0680 (4)	0.0576 (3)	0.0639 (4)	−0.0096 (2)	−0.0027 (3)	−0.0157 (2)
N1	0.0444 (8)	0.0407 (7)	0.0364 (7)	−0.0022 (6)	0.0051 (6)	0.0000 (6)
C2	0.0390 (8)	0.0434 (9)	0.0349 (8)	0.0031 (7)	0.0055 (6)	0.0003 (7)
C3	0.0438 (9)	0.0498 (10)	0.0381 (9)	0.0080 (8)	0.0052 (7)	−0.0042 (7)
C4	0.0973 (18)	0.0617 (13)	0.0363 (10)	0.0043 (12)	0.0063 (10)	0.0000 (9)
C6	0.0610 (11)	0.0502 (11)	0.0442 (10)	0.0033 (9)	0.0124 (9)	0.0077 (8)
C7	0.0411 (9)	0.0429 (9)	0.0377 (8)	0.0044 (7)	0.0072 (7)	0.0016 (7)
C8	0.0365 (8)	0.0415 (9)	0.0395 (9)	0.0038 (7)	0.0057 (7)	0.0010 (7)
C9	0.0436 (9)	0.0422 (9)	0.0470 (10)	−0.0018 (7)	0.0058 (7)	0.0016 (7)
C10	0.0415 (9)	0.0449 (9)	0.0476 (10)	0.0011 (7)	0.0025 (7)	−0.0079 (8)
C11	0.0431 (9)	0.0549 (10)	0.0372 (9)	0.0034 (8)	0.0020 (7)	−0.0021 (8)
C12	0.0445 (9)	0.0498 (10)	0.0372 (9)	0.0004 (8)	0.0062 (7)	0.0034 (7)
C13	0.0341 (8)	0.0410 (8)	0.0392 (9)	0.0023 (6)	0.0053 (6)	−0.0003 (7)
C14	0.0495 (10)	0.0526 (10)	0.0400 (9)	0.0051 (8)	0.0028 (7)	−0.0096 (8)
C15	0.0501 (11)	0.0473 (10)	0.0514 (11)	−0.0014 (8)	0.0040 (8)	−0.0144 (9)
N16	0.0836 (14)	0.0497 (10)	0.0637 (12)	−0.0114 (9)	0.0072 (10)	−0.0025 (9)
C17	0.0872 (16)	0.0681 (15)	0.0516 (12)	−0.0068 (12)	0.0053 (11)	−0.0163 (11)
N18	0.159 (3)	0.124 (2)	0.0595 (14)	−0.030 (2)	−0.0041 (15)	−0.0360 (15)
C5A	0.047 (3)	0.040 (3)	0.040 (3)	0.018 (2)	0.0090 (18)	0.0046 (18)
C5B	0.362 (17)	0.242 (13)	0.047 (4)	−0.241 (13)	−0.046 (7)	0.048 (6)

### Geometric parameters (Å, °)

**Table:**

Cl1—C10	1.7498 (18)	C6—H6D	0.9700
N1—C13	1.371 (2)	C7—C8	1.421 (2)
N1—C2	1.392 (2)	C8—C9	1.407 (2)
N1—H1	0.94 (3)	C8—C13	1.418 (2)
C2—C7	1.395 (2)	C9—C10	1.371 (3)
C2—C3	1.431 (2)	C9—H9	0.9300
C3—C14	1.368 (3)	C10—C11	1.404 (3)
C3—C4	1.507 (3)	C11—C12	1.375 (3)
C4—C5B	1.354 (9)	C11—H11	0.9300
C4—C5A	1.538 (6)	C12—C13	1.401 (2)
C4—H4A	0.9700	C12—H12	0.9300
C4—H4B	0.9700	C14—C17	1.431 (3)
C4—H4C	0.9700	C14—C15	1.433 (3)
C4—H4D	0.9700	C15—N16	1.145 (3)
C6—C7	1.493 (2)	C17—N18	1.144 (3)
C6—C5A	1.509 (7)	C5A—H5A	0.9700
C6—C5B	1.443 (8)	C5A—H5B	0.9700
C6—H6A	0.9700	C5B—H5C	0.9700
C6—H6B	0.9700	C5B—H5D	0.9700
C6—H6C	0.9700		
			
C13—N1—C2	108.16 (14)	C7—C6—H6D	109.5
C13—N1—H1	122.9 (15)	C5A—C6—H6D	91.2
C2—N1—H1	128.7 (15)	C5B—C6—H6D	109.5
N1—C2—C7	109.17 (15)	H6A—C6—H6D	125.2
N1—C2—C3	127.82 (16)	H6B—C6—H6D	20.8
C7—C2—C3	123.01 (16)	H6C—C6—H6D	108.1
C14—C3—C2	125.59 (17)	C2—C7—C8	107.08 (15)
C14—C3—C4	119.64 (17)	C2—C7—C6	123.36 (16)
C2—C3—C4	114.75 (17)	C8—C7—C6	129.57 (17)
C5B—C4—C3	119.5 (4)	C9—C8—C13	119.94 (15)
C5B—C4—C5A	20.5 (8)	C9—C8—C7	133.40 (16)
C3—C4—C5A	114.5 (3)	C13—C8—C7	106.66 (15)
C5B—C4—H4A	120.7	C10—C9—C8	117.49 (16)
C3—C4—H4A	108.6	C10—C9—H9	121.3
C5A—C4—H4A	108.6	C8—C9—H9	121.3
C5B—C4—H4B	88.6	C9—C10—C11	122.64 (17)
C3—C4—H4B	108.6	C9—C10—Cl1	119.80 (15)
C5A—C4—H4B	108.6	C11—C10—Cl1	117.56 (14)
H4A—C4—H4B	107.6	C12—C11—C10	120.82 (17)
C5B—C4—H4C	107.4	C12—C11—H11	119.6
C3—C4—H4C	107.4	C10—C11—H11	119.6
C5A—C4—H4C	125.9	C11—C12—C13	117.80 (16)
H4A—C4—H4C	87.5	C11—C12—H12	121.1
H4B—C4—H4C	22.3	C13—C12—H12	121.1
C5B—C4—H4D	107.4	N1—C13—C12	129.75 (16)
C3—C4—H4D	107.4	N1—C13—C8	108.94 (15)
C5A—C4—H4D	91.7	C12—C13—C8	121.31 (16)
H4A—C4—H4D	20.4	C3—C14—C17	120.97 (19)
H4B—C4—H4D	125.3	C3—C14—C15	125.38 (16)
H4C—C4—H4D	107.0	C17—C14—C15	113.64 (19)
C7—C6—C5A	109.1 (3)	N16—C15—C14	176.9 (2)
C7—C6—C5B	110.8 (3)	N18—C17—C14	178.6 (3)
C5A—C6—C5B	21.2 (8)	C6—C5A—C4	111.1 (3)
C7—C6—H6A	109.9	C6—C5A—H5A	109.4
C5A—C6—H6A	109.9	C4—C5A—H5A	109.4
C5B—C6—H6A	90.1	C6—C5A—H5B	109.4
C7—C6—H6B	109.9	C4—C5A—H5B	109.4
C5A—C6—H6B	109.9	H5A—C5A—H5B	108.0
C5B—C6—H6B	125.5	C4—C5B—C6	127.8 (6)
H6A—C6—H6B	108.3	C4—C5B—H5C	105.3
C7—C6—H6C	109.5	C6—C5B—H5C	105.3
C5A—C6—H6C	127.2	C4—C5B—H5D	105.3
C5B—C6—H6C	109.5	C6—C5B—H5D	105.3
H6A—C6—H6C	21.5	H5C—C5B—H5D	106.0
H6B—C6—H6C	89.0		
			
C13—N1—C2—C7	0.50 (19)	Cl1—C10—C11—C12	179.78 (14)
C13—N1—C2—C3	−178.87 (16)	C10—C11—C12—C13	−0.2 (3)
N1—C2—C3—C14	−0.5 (3)	C2—N1—C13—C12	179.17 (17)
C7—C2—C3—C14	−179.80 (17)	C2—N1—C13—C8	−0.47 (18)
N1—C2—C3—C4	−178.92 (18)	C11—C12—C13—N1	−179.40 (17)
C7—C2—C3—C4	1.8 (3)	C11—C12—C13—C8	0.2 (2)
C14—C3—C4—C5B	175.1 (9)	C9—C8—C13—N1	179.52 (14)
C2—C3—C4—C5B	−6.4 (9)	C7—C8—C13—N1	0.26 (18)
C14—C3—C4—C5A	152.7 (3)	C9—C8—C13—C12	−0.2 (2)
C2—C3—C4—C5A	−28.8 (3)	C7—C8—C13—C12	−179.41 (15)
N1—C2—C7—C8	−0.33 (19)	C2—C3—C14—C17	179.43 (19)
C3—C2—C7—C8	179.07 (15)	C4—C3—C14—C17	−2.2 (3)
N1—C2—C7—C6	179.84 (16)	C2—C3—C14—C15	−1.2 (3)
C3—C2—C7—C6	−0.8 (3)	C4—C3—C14—C15	177.1 (2)
C5A—C6—C7—C2	26.1 (3)	C3—C14—C15—N16	−173 (4)
C5B—C6—C7—C2	3.7 (8)	C17—C14—C15—N16	6(4)
C5A—C6—C7—C8	−153.6 (2)	C3—C14—C17—N18	172 (14)
C5B—C6—C7—C8	−176.1 (8)	C15—C14—C17—N18	−7(14)
C2—C7—C8—C9	−179.07 (17)	C7—C6—C5A—C4	−50.9 (4)
C6—C7—C8—C9	0.7 (3)	C5B—C6—C5A—C4	47.5 (11)
C2—C7—C8—C13	0.05 (18)	C5B—C4—C5A—C6	−53.9 (13)
C6—C7—C8—C13	179.86 (18)	C3—C4—C5A—C6	55.0 (4)
C13—C8—C9—C10	0.1 (2)	C3—C4—C5B—C6	11 (2)
C7—C8—C9—C10	179.11 (17)	C5A—C4—C5B—C6	92 (2)
C8—C9—C10—C11	−0.1 (3)	C7—C6—C5B—C4	−9.1 (19)
C8—C9—C10—Cl1	−179.71 (12)	C5A—C6—C5B—C4	−99 (2)
C9—C10—C11—C12	0.1 (3)		

### Hydrogen-bond geometry (Å, °)

**Table:**

*D*—H···*A*	*D*—H	H···*A*	*D*···*A*	*D*—H···*A*
N1—H1···N16	0.94 (3)	2.60 (3)	3.373 (2)	139.5 (19)
N1—H1···N16^i^	0.94 (3)	2.27 (3)	3.099 (3)	147 (2)
C11—H11···N18^ii^	0.93	2.48	3.352 (3)	156.

Symmetry codes:  (i) −*x*, −*y*, −*z*+1; (ii) *x*+1/2, −*y*+1/2, *z*+1/2.
